# Improving early childhood nutrition practices through parents’ evening forums in rural Rwanda

**DOI:** 10.1017/S1368980025100803

**Published:** 2025-08-12

**Authors:** Fabien Nsanzabera, Evangeline Irakoze, Alexis Manishimwe, Jean Bosco Nsengiyumva, Aimable Mwiseneza, Emmanuel Ntakirutimana, Fabien Nkurikiyimana

**Affiliations:** Department of Education in Sciences, Faculty of Education, University of Technology and Arts of Byumba (UTAB), Gicumbi, Rwanda

**Keywords:** Early childhood nutrition, Community-based education, Malnutrition prevention, Parents’ evening forums, Rural health education

## Abstract

**Objective:**

This study aimed to evaluate early childhood nutrition knowledge and practices in Gicumbi District, Rwanda, and assess the potential of parents’ evening forums as platforms for community-based nutrition education.

**Design:**

This study employed a mixed-methods design incorporating structured questionnaires (quantitative) and focus group discussions and interviews (qualitative). Quantitative data were analysed using descriptive statistics and Spearman’s rank correlation to explore associations among participation, knowledge application and access barriers. Thematic analysis was applied to qualitative data to capture contextual insights and educational preferences.

**Setting:**

The study was conducted in Gicumbi District, a rural region in northern Rwanda, characterised by high malnutrition rates.

**Participants:**

523 participants: 471 household heads completed questionnaires; 52 took part in focus group discussions and interviews.

**Results:**

The study revealed substantial knowledge gaps, with only 46 % of participants aware of the symptoms of malnutrition and just 32 % identifying nutrient-rich complementary foods. Despite 68 % of participants reporting social connection as a key motivator for joining parents’ evening forums, logistical challenges such as time and travel barriers were cited by 41 % as constraints. Lectures were the most preferred teaching method (78 %), followed by cooking demonstrations (56 %). Qualitative findings emphasised the importance of local relevance, peer support and interactive learning for fostering participation and knowledge retention.

**Conclusions:**

Parents’ evening forums represent a viable and contextually appropriate platform for delivering early childhood nutrition education. Their expansion, alongside the integration of digital tools and tailored, experiential teaching approaches, could strengthen community engagement and address persistent malnutrition challenges in Rwanda and comparable settings.

Early childhood is a crucial time for cognitive and growth development, and during this time, long-term health outcomes are greatly influenced by dietary status. The significance of sufficient nutrition throughout the first 1000 days of life is highlighted by the quick physical and neurological development that takes place during this time^(1)^. Proper nutrition is essential for preventing stunting, wasting and other forms of malnutrition that can lead to irreversible developmental delays and increase the risk of chronic diseases later in life^(2)^. As part of an all-encompassing plan to enhance child nutrition, the WHO and UNICEF promote ideal infant and young child feeding practices globally. The first 6 months of exclusive breastfeeding, continuing to breastfeed for at least 2 years and introducing safe and nutritionally appropriate complementary foods on schedule beginning at 6 months are some examples of these best practices^(3)^. Unfortunately, many regions of the world continue to adhere to these recommendations below optimal levels, especially in low- and middle-income countries, where socio-economic barriers, cultural beliefs and restricted access to healthcare services impede the adoption of recommended feeding practices^(4)^.

Numerous interrelated factors, such as maternal education, socio-economic status, access to healthcare and cultural norms, influence how infants and little children are fed. These elements influence children’s food patterns, which in turn affects their short-term nutrient intake and long-term dietary habits^(5)^. For example, better educated mothers are more likely to follow guidelines for breastfeeding practices and to provide their child with a more diversified food during the supplemental feeding time^(6)^. On the other hand, in environments with limited resources, a lack of understanding about appropriate feeding techniques combined with financial limitations frequently results in a reliance on foods that are undernourished, prolonging cycles of ill health and malnutrition^(7,8)^. An estimated 144 million children under five experience stunting, and 47 million are afflicted by wasting, demonstrating that malnutrition is a continued worldwide health issue^(9)^. These disorders are especially common in sub-Saharan Africa, where poor nutritional outcomes are caused by socio-economic difficulties, food shortages and restricted access to healthcare^(10)^. Public health interventions are made more difficult by the emergence of the double burden of malnutrition, which is defined by the coexistence of obesity and undernutrition within the same population^(11)^.

The high rate of malnutrition among children under five in Rwanda continues to be a serious problem. Even while maternal health and infant mortality have significantly improved, 33 % of Rwandan children are stunted, and the percentage rises even worse in rural areas. Specifically, in Gicumbi district, stunting affects approximately 41 % of children under five, significantly higher than the national average, highlighting the district’s heightened vulnerability^(12)^. These issues are clearly demonstrated in the Gicumbi district, where socio-economic inequalities, a lack of access to a variety of nutrient-rich foods and a lack of mother education regarding appropriate newborn feeding practices all lead to chronic malnutrition^(13)^. In Rwanda, a community-based programme known as the parents’ evening forum (*Umugoroba w’Ababyeyi*) has long addressed societal concerns like gender equality, family harmony and cleanliness^(14)^. Its potential as a venue for nutrition teaching has not been thoroughly investigated, though. Incorporating nutrition education into this well-established forum offers a fresh way to combating malnutrition, since community participation and mother education play a critical role in influencing dietary choices and enhancing child nutrition^(15,16)^.

The purpose of this study is to determine how well nutrition education can be incorporated into the parents’ evening forum to improve early dietary behaviours and lower the rate of malnutrition in rural Rwanda. In particular, it evaluates parents’ understanding of malnutrition, looks at ways to convey nutrition facts clearly and pinpoints the best ways to teach in order to boost community engagement and retention of important concepts. This aligns with Rwanda’s broader nutrition policy objectives and contributes to evidence-based strategies that could be adopted across similar rural communities. The study provides a culturally appropriate and long-lasting paradigm for enhancing nutritional outcomes in situations with limited resources by utilising an already-existing community structure. The findings from the research are anticipated to add to the global conversation about community-based nutrition interventions by offering perceptions that might guide comparable initiatives in other low-resource areas^(17)^. Additionally, the inclusion of nutrition education in the parents’ evening forum is in line with the most recent guidelines from the WHO and UNICEF^(18)^. These organisations support multi-sectoral approaches to combating malnutrition, especially through community-based initiatives that reinforce the international strategy for feeding infants and young children^(16)^. If the integration of nutrition education into the parents’ evening forum proves effective in Gicumbi district, the model could be expanded throughout Rwanda and in comparable regions across sub-Saharan Africa, providing a culturally embedded strategy for enhancing child nutrition and lowering morbidity and mortality from malnutrition on a broader scale^(19,20)^.

## Materials and methods

The study was conducted in Gicumbi district, Rwanda, involving 523 participants. These participants included 471 heads of households, selected from attendees of the parents’ evening forum across three sectors of Gicumbi district, with a gender distribution of 188 men and 283 women. Strata were defined by geographic location (sector), gender and age group (young parents < 30 years, middle-aged 30–50 years and older > 50 years), to ensure diverse demographic representation across the district. Additionally, the study included forty-five target group members and seven key informants to provide comprehensive insights. A stratified random sampling method was employed to select the 471 heads of households, ensuring representation from the three sectors of the district and considering various demographic factors. The sampling frame consisted of lists of households with children under 5 years who regularly attended the parents’ evening forum, obtained from sector-level community health workers. From these lists, households were randomly selected proportionally from each sector. The forty-five target group members were selected for their involvement in community nutrition programmes, while the seven key informants were chosen based on their expertise in health and nutrition.

This study employed a convergent parallel mixed-methods design to collect and analyse both quantitative and qualitative data. This approach was chosen to triangulate findings and enhance the validity of the results by allowing the integration of statistical trends with community perspectives. Data collection involved the use of structured questionnaires, focus group discussions (FGD) and semi-structured interviews. The structured questionnaires were administered face-to-face by trained data collectors to all 471 heads of households. These questionnaires included a mix of closed-ended and open-ended questions designed to assess participants’ knowledge of malnutrition, sources of nutrition information and preferences for nutrition education. Data were collected using paper-based forms between 3 May and 20 June 2024. Each questionnaire took approximately 30–45 min to complete. FGD were held with the forty-five target group members to delve deeper into community perceptions and challenges related to malnutrition and nutrition education. There were five FGD, each composed of 8–10 participants, grouped by sector and gender to ensure homogeneity and facilitate open discussion. The FGD and interviews were conducted in Kinyarwanda (native language), audio-recorded with consent, transcribed verbatim and translated into English. The seven key informants provided expert insights through semi-structured interviews, which also served to validate the data collected from the households and target group members. Interviews were conducted face-to-face in private settings to ensure confidentiality, and they lasted between 30 and 45 min. Quantitative and qualitative components were implemented concurrently, and results were integrated during the interpretation phase. Qualitative findings were used to contextualise quantitative results and offer explanatory depth; for instance, FGD highlighted reasons behind low awareness levels found in the survey.

Data analysis was conducted using SPSS for the quantitative data, with descriptive statistics (such as frequencies and percentages) calculated to summarise the findings. Correlation analysis was conducted using Spearman’s correlation coefficient to assess the strength and direction of relationships between continuous or ordinal variables, such as age, education level and malnutrition knowledge scores. For categorical variables such as gender, point-biserial correlation was used. Significance was evaluated at the *P* < 0·05 level. This analytical method allowed for a more nuanced understanding of how demographic characteristics influenced nutrition knowledge. The qualitative data from the FGD and interviews were analysed thematically to identify key themes related to knowledge gaps, barriers and educational preferences. Following Braun and Clarke’s (2006) six-step framework, transcripts were independently coded by two researchers. Emergent codes were discussed and grouped into overarching themes through iterative consensus meetings. Themes were derived inductively and validated through cross-case comparisons. The study adhered to principles of phenomenological inquiry, focusing on participants’ lived experiences and perceptions regarding malnutrition and nutrition education^(21)^. To establish rigour, various trustworthiness strategies were applied. For qualitative data, credibility was ensured through triangulation (interviews and FGD), member checking was conducted for two FGD and peer debriefing supported dependability. Confirmability was enhanced by maintaining an audit trail. For the quantitative component, the questionnaire was pretested on thirty individuals from a neighbouring sector to ensure clarity and reliability. Generalisability was supported through stratified sampling. The overall mixed-methods validity was enhanced through convergence and confirmation of data sources.

Ethical considerations were rigorously followed, with informed consent obtained from all participants. The study protocol was approved by an ethical review board, ensuring the protection of participant confidentiality and rights throughout the research process. All data collectors were university-trained in education studies and received 2 days of training on research ethics and data collection procedures prior to fieldwork. This methodology provided a comprehensive approach to understanding community knowledge of malnutrition and identifying effective strategies for integrating nutrition education into parents’ evening forums.

## Results and Discussions

### Knowledge on malnutrition

Millions of people worldwide are still impacted by malnutrition, which is especially bad in low- and middle-income nations. Nearly half of the study’s participants (48 %) showed a moderate level of knowledge regarding malnutrition, showing a general but insufficient grasp of the problem (Table 1). This is consistent with global trends, where large segments of the populace, especially in places with little access to thorough health education, are still only marginally aware of the complexity of malnutrition despite expanded awareness campaigns^(22)^. The discovery that 18 % of participants knew very little to nothing about malnutrition draws attention to a crucial knowledge gap that is corresponding with issues facing the world today (Table 1). Malnutrition persists in many areas, especially in rural and underprivileged groups, due to a lack of access to trustworthy nutrition information and education. This emphasises the requirement for specialised educational initiatives that offer knowledge that is both useful and actionable in addition to increasing awareness^(23)^. The issue appears to be one of both awareness and access to education, as evidenced by the fact that 36 % of respondents had never received any formal nutrition education (Table 1). This is consistent with a problem that exists across the globe^(24)^.

**Table 1.** Current knowledge of community on malnutrition

Statement	Category	*n*	%
Familiarity with the concept of malnutrition	Moderately familiar	251	48
	Highly familiar	178	34
	Unfamiliar	94	18
Prior education on nutrition	Yes	335	64
	No	188	36
Perception of malnutrition as a community issue	No	146	28
	Yes	136	26
Perceived contributors to child malnutrition	Illness-related nutrient absorption issues	115	22
	Economic hardship	94	18
	Cultural influences	10	2
	Poor hygiene and sanitation	10	2
	Limited awareness on nutrition	10	2
Personal or observed experiences with malnutrition	Yes	335	64
	No	188	36
Self-assessed nutrition knowledge	Moderate	230	44
	High	146	28
	Lacking	146	28
Perceived key causes of child malnutrition	Inadequate access to nutritious food	502	96
	Insufficient food intake	481	92
Typical sources of nutrition information	Non-governmental organisations	439	84
	Online platforms/websites	262	50
	Government or healthcare sectors	251	48
	Books/literature	178	34
	Nutritionists/dietitians	94	18
Involvement in nutrition or health-related programmes	Yes	366	70
	No	157	30

*Source:* This study.

The findings highlight how crucial it is to enhance instructional strategies and educational outreach, especially through neighbourhood-based programmes like the parents’ evening forums. These forums have the potential to be extremely helpful in closing the education gap by offering easily available and contextually appropriate knowledge. This strategy is in line with worldwide best practices, which support community-driven health education as a way to provide people with the information they need to prevent and treat malnutrition^(25)^. This approach is also directly aligned with Rwanda’s National Strategy for Transformation (NST2), particularly Pillar 1 (Economic Transformation) and Pillar 3 (Transformational Governance), which aim to address malnutrition through integrated community health programmes that emphasise local engagement and education. These programmes are designed to empower communities to take charge of their nutritional health, thereby contributing to the SDG, particularly Goal 2: Zero Hunger^(26)^. There is ample evidence worldwide between the frequency of malnutrition and knowledge levels. The fact that 64 % of research participants had either personally experienced malnutrition or knew someone who had highlights the problem’s broad character and the demand for focused solutions. The overlap in knowledge levels, where 44 % of respondents said they understood things fairly and 28 % said they knew nothing at all or had excellent knowledge (Table 1), highlights the differences in health literacy that are prevalent throughout the world. Effective malnutrition prevention and treatment depends on addressing these gaps^(27)^. Additionally, focus group discussions highlighted that parents often felt overwhelmed by the abundance of information available online, leading to confusion about reliable sources and practical steps they could take.

Global understandings of the underlying causes of malnutrition are further supported by the study’s findings, which show that nearly all respondents consider access to nutrient-dense food (96 %) and an adequate food intake (92 %) to be crucial issues (Table 1). These findings highlight the significance of programmes aimed at ensuring food security, which are vital both domestically in Rwanda and globally. Rwanda’s commitment to achieving the SDG includes enhancing food security through sustainable agricultural practices and community-based interventions that align with the NST2 framework^(26)^. International organisations like the FAO and the WHO recommend strategies like the promotion of community gardens, support for local agriculture and initiatives aimed at improving access to fresh produce as effective measures to combat food insecurity and malnutrition^(28)^. A lack of expert assistance is seen in the reliance of 84 % of respondents on charity groups and 50 % of respondents on websites to obtain information relevant to nutrition, whereas only 18 % of respondents consulted specialist nutritionists (Table 1). This phenomenon is observed throughout the world in regions where communities frequently rely on non-expert sources for information and have limited access to professional nutritionists. As this study suggests, increasing the number of certified nutritionists involved in educational activities would be in line with international guidelines to guarantee accurate, culturally relevant and community-specific nutrition education^(29)^. Ultimately, the government plays a crucial role in disseminating trustworthy and easily available dietary information. The most vulnerable communities can be reached by educational programmes through government-led initiatives in rural and peri-urban areas, where resources and infrastructure may be scarce. This strategy is vital for Rwanda and is supported under NST2 Priority Area 4, which focuses on strengthening citizen participation and community health^(26)^, as well as national SDG targets such as Goal 2 (Zero Hunger), Goal 3 (Good Health and Well-being) and Goal 4 (Quality Education)^(30)^. Governments can contribute to the global fight against hunger by improving the efficacy of nutrition education initiatives by incorporating community feedback and tailoring strategies to local contexts^(31)^.

### Strategies to integrate nutrition education into parents’ evening forums

Table 2 indicates that interactive, hands-on learning is greatly preferred by 80 % of respondents, indicating that educational programmes should be developed to be engaging and dynamic. This result is consistent with international trends in education, which highlight experiential learning as a useful strategy for information retention and behaviour modification^(32)^. While a third of participants may be attracted by a flexible schedule, over half (54 %) are keen to attend programmes in the evening. This is in line with research from other countries that indicates that flexible learning schedules are essential to boosting enrolment in adult education programmes, particularly in areas where families have varying work schedules and responsibilities to their children^(33)^. Twelve percent of respondents expressed no interest, indicating that barriers or alternative methods of reaching them need to be addressed. Furthermore, the findings indicated that the most popular themes were child nutrition (72 %) and balanced meals (56 %), suggesting that these topics ought to be prioritised in instructional materials. The focus on child nutrition around the world is a reflection of the urgent need to address early eating habits in order to prevent long-term health problems including obesity and stunting^(34)^. Addressing child nutrition is particularly critical for Rwanda, which has made reducing childhood stunting a key target under both NST2^(26)^ and SDG 2 (Zero Hunger)^(30)^. The global trend towards skill-based nutrition education, which is acknowledged for its influence on dietary behaviour and food choices^(35)^, is further supported by the fact that almost 50 % of respondents find value in practical advice on meal preparation and cooking. This is supported by qualitative feedback, which revealed that parents preferred interactive sessions where they could engage directly with nutrition experts, ask questions and participate in hands-on activities.

**Table 2.** Integration of nutrition education into parents’ evening forum

Statement	Category	*n*	%
Preferred timing for attending educational sessions	Morning	356	68
	Evening	167	32
Learning style preference (traditional *v*. interactive)	interactive/hands-on approaches preferred	418	80
	Prefer traditional approach	94	18
Likelihood of attending sessions offering incentives	Yes	366	70
	No	157	30
Willingness to attend evening nutrition sessions for parents	Definitely willing	282	54
	Maybe, depending on timing	178	34
	Not interested	63	12
Topics of interest for nutrition sessions	Nutritional needs of children	377	72
	Balanced diet education	293	56
	Recognising malnutrition symptoms	282	54
	Cooking and food preparation guidance	209	40
Barriers to attending evening nutrition education	Family obligations	335	64
	Transport challenges	262	50
	Work commitments	251	48
	Financial limitations	209	40
	Health-related concerns	84	16
	Cultural or societal norms	21	4
	Lack of awareness	21	4
Challenges in accessing nutrition education/resources	Yes	397	76
	No	126	24

*Source:* This study.

However, family responsibilities (64 %) and transportation issues (50 %) provide the largest barriers to attending the parents’ evening forums (Table 2). Raising these factors could result in higher attendance numbers. Providing day care during the parents’ evening forum sessions could be helpful in light of family duties. This strategy has been effectively applied in a number of worldwide community education initiatives, greatly increasing the participation rates of parents, especially mothers, in these programmes^(36)^. As evidenced by international initiatives that emphasise placing community programmes within walking distance of participants’ houses to minimise logistical obstacles, holding the events in easily accessible locations could also help with transportation-related concerns^(37)^. While health issues and cultural norms only affect a smaller portion of the population, financial constraints and work commitments are still significant obstacles. Additionally, Table 2 data revealed that 76 % of participants had trouble obtaining nutrition-related resources or information, highlighting the critical need for an intervention through the parents’ evening forum. Financial obstacles to health education have been successfully removed globally by integrating free or inexpensive educational programmes that are backed by local governments or international non-governmental organisations^(38)^. To help with budgetary constraints, the parents’ evening forum might be provided as a community service at no cost, and it could be scheduled at many times, including on weekends, to meet different work schedules. Furthermore, the parents’ evening forum educational programme’s integration of technology, which allows for remote access to educational resources via websites, mobile apps and virtual meetings, reflects global trends towards e-learning and mHealth initiatives. Leveraging (Information and Communication Technology (ICT) to deliver nutrition education would complement Rwanda’s digital ambition under NST2^(26)^ and support SDG 9 (Industry, Innovation and Infrastructure)^(30)^, fostering inclusive access to health knowledge across the country. These initiatives have proven particularly effective in reaching marginalised populations in both urban and rural settings^(39)^.

### Preference of teaching techniques for parents’ evening forum

The parents’ evening forum attendees provided an explanation of their decision to participate (Table 3). Social connection is the biggest motivator (68 %), followed by goals (44 %) and personal development (58 %). This is in line with global trends in community-based education, where participation is frequently driven by social interaction and personal growth, especially in adult learning situations^(40)^. Curiosity and a sense of accomplishment are noteworthy but less significant. To maximise participation, programmes should incorporate social components and opportunities for personal development. Empathy and opportunity for personal development are important components of educational programmes that have been found to dramatically increase engagement and retention^(41)^. Nearly equal numbers of people (54 %) and those (46 %) who have not attended evening forums suggest that there may be an opportunity to increase participation through targeted outreach and the removal of barriers (Table 3). This result is in line with findings from other countries that point to the necessity of focused outreach and removing obstacles including time, place and perceived importance in order to increase participation in community education programmes^(42)^.

**Table 3.** Teaching strategies on knowledge of nutrition through parents’ evening forum

Statement	Category	*n*	%
Motivations for participating in educational programmes	Social engagement	356	68
	Self-improvement	303	58
	Achieving personal objectives	230	44
	Interest or curiosity	146	28
	Feeling of achievement	146	28
Prior attendance at evening educational sessions	Yes	282	54
	No	241	46
Preferred teaching methods for evening malnutrition sessions	Traditional lectures	408	78
	Cooking-based demonstrations	293	56
	Participatory activities	241	46
	Role-playing exercises	157	30
	Group-based discussions	136	26
Likelihood of applying knowledge from sessions in daily life	Moderately likely	272	52
	Highly likely	157	30
	Unlikely	94	18

*Source:* This study.

The primary cause of social contact could be taken into consideration while creating educational programmes with social interaction elements, including group projects or neighbourhood gatherings. Studies conducted throughout the world show how beneficial it is to include social contact in educational programmes in order to promote a feeling of community and shared learning. This is especially true in situations where health education is being taught^(43)^. The findings also show that 78 % of respondents thought lectures were the most effective method, with interactive activities coming in second (46 %) and cooking demonstrations in third (56 %) (Table 3). This aligns with global best practices that support a combination of interactive, hands-on learning and traditional lectures to accommodate different learning styles and improve retention of information^(44)^. Group conversations and role-playing are useful but less favoured. A variety of preferences can be accommodated by employing a mixed-method approach. A mixed-method approach is becoming more and more popular around the world as a way to guarantee that students comprehend and apply the material being taught by blending lectures with interactive and participatory approaches^(32)^. Lectures with cooking demonstrations may fulfil the highest needs; interactive exercises and role-playing engage participants and make learning more dynamic, and group discussions can foster peer learning and support. This finding is in accordance with qualitative insights indicated that parents often felt lectures alone were insufficient. Many expressed a desire for a more engaging format that combined information delivery with practical application.

Moreover, a majority of participants (52 %) express at least a moderate inclination to use the knowledge they have acquired, with 30 % indicating a high likelihood of doing so. This is in line with research from across the globe that indicates practical, hands-on learning methods greatly raise the possibility that participants will use what they have learned in authentic settings^(45)^. Nevertheless, 18 % of participants are unlikely to use what they learn, indicating the need for strategies to increase their applicability in real-world situations. It is imperative to close this gap by creating context-specific, implementable learning strategies, which are in line with international educational approaches that stress the value of practical relevance in health education^(46)^. The effectiveness of nutrition education programmes through the parents’ evening forum might be highlighted by the implementation of mechanisms for continuing participant input to refine and improve educational offerings.

### Critical insights into community participation and nutritional education strategies

The analysis of correlations among variables influencing participation in nutrition education programmes reveals several critical insights with direct relevance to national and global development goals (Table 4). A strong positive correlation was observed between individuals’ willingness to attend nutrition education sessions and their likelihood of applying acquired knowledge in daily life (ρ = 0·62, *P* < 0·01). This suggests that motivation to attend is not passive but translates into actionable health behaviour changes. Similarly, individuals with a history of participating in evening education sessions were significantly more willing to attend future sessions (ρ = 0·44) and showed a higher likelihood of applying the learned knowledge (ρ = 0·37), emphasising the cumulative impact of educational exposure. Motivational factors such as social interaction (ρ = 0·38) and preference for interactive teaching methods like cooking demonstrations (ρ = 0·41) also showed positive associations with session attendance. These findings highlight the importance of designing community-based nutrition interventions that are both experiential and socially engaging, aligning with Rwanda’s NST2 Priority Area 4, which seeks to improve early childhood development and reduce malnutrition through inclusive, community-driven health promotion strategies^(26)^. Furthermore, the data support the idea that citizen participation in health initiatives is enhanced when the learning experience is collaborative and directly applicable to real-life settings. Conversely, barriers such as transportation difficulties, family obligations and financial constraints were negatively correlated with both attendance and knowledge application (ρ = –0·35 and –0·28, respectively). This underscores the need for structural and policy interventions to reduce access-related inequities, particularly in underserved and rural communities. These findings resonate with SDG 2 (Zero Hunger) and SDG 3 (Good Health and Well-being), where access to accurate nutrition education is fundamental in empowering communities to make informed dietary decisions. Moreover, the preference for interactive education also aligns with SDG 4 (Quality Education), which advocates for inclusive and equitable lifelong learning opportunities^(30)^. Collectively, the correlation analysis suggests that motivation, prior engagement and teaching methodology are significant predictors of successful participation and knowledge retention in nutrition programmes. As Rwanda continues to implement the NST2 framework and align with the SDG, enhancing the design, delivery and accessibility of nutrition education will be crucial in ensuring sustainable improvements in public health outcomes.

**Table 4.** Spearman’s rank correlation coefficients among variables influencing participation and engagement in nutrition education programmes

Variables	Willingness to attend	Apply knowledge	Access challenges	Attended before	Motivation: social interaction	Teaching: cooking demos
Willingness to attend	1·00	0·62**	−0·35**	0·44**	0·38**	0·41**
Apply knowledge	0·62**	1·00	−0·28*	0·37**	0·33**	0·36**
Access challenges	−0·35**	−0·28*	1·00	−0·31*	−0·22*	−0·25*
Attended before	0·44**	0·37**	−0·31*	1·00	0·30**	0·29**
Motivation: social interaction	0·38**	0·33**	−0·22*	0·30**	1·00	0·26*
Teaching: cooking demonstrations	0·41**	0·36**	−0·25*	0·29**	0·26*	1·00

*Source:* This study.

Legends: (1) All variables are coded: Yes = 1, No = 0. Ordinal responses are numerically scaled. (2) Spearman’s ρ is used due to the ordinal/binary nature of the data. (3) *P < 0·05, **P < 0·01 indicate statistically significant correlations. (4) *n* 523 reflects the total respondents. (5) Positive coefficients reflect supportive associations; negative coefficients indicate inverse relationships.

### Strengths and limitations of the study

This study has several strengths that enhance its contributions to the field. First, it engaged a diverse participant pool from various backgrounds, which improves the generalisability of the findings across different community settings. Additionally, the use of a mixed-methods approach allowed for a comprehensive understanding of participants’ preferences and motivations, enriching the data interpretation. The findings also align with global trends in community-based education, reinforcing their relevance and applicability, particularly in adult learning contexts. Furthermore, the emphasis on practical, hands-on learning methods reflects current best practices in health education, promoting the real-world applicability of the knowledge gained. Lastly, the inclusion of mechanisms for ongoing participant feedback can help refine and improve educational offerings over time, ensuring they remain relevant to community needs.

However, the study also has limitations that should be acknowledged. The overall sample size, while diverse, may limit the statistical power of the findings, potentially affecting the robustness of the conclusions drawn. Additionally, the reliance on self-reported data may introduce biases, as participants might overestimate their motivations or likelihood of applying the knowledge gained. The geographic scope of the study was also limited to a specific region, which may restrict the applicability of the findings to other areas with different cultural or educational contexts. There is also the potential for response bias, as participants who chose to attend the forum may differ in characteristics or motivations from those who did not participate. Finally, due to its cross-sectional nature, the study primarily assessed immediate reactions and preferences without long-term follow-up to evaluate the sustained impact of the educational programmes on participants’ behaviour and knowledge application.

### Conclusion and future perspectives

This study highlights the critical importance of early childhood nutrition, which is a pressing issue affecting children globally, and identifies critical knowledge gaps in the Gicumbi district of Rwanda. Although there is a certain level of awareness regarding malnutrition, a substantial portion of the population lacks knowledge on practical preventive measures, which is a situation that is similar to that of other low- and middle-income nations. As community-based platforms, parents’ evening forums present a promising model for closing these gaps through focused, practical nutrition education that may be adapted for application in other similar contexts across the globe. The results of this study demonstrate a strong preference for experiential learning and flexible scheduling, which is consistent with global best practices in adult education. These strategies should improve participation and knowledge application in Rwanda and similar contexts around the world. To maximise involvement and impact, common impediments including family obligations and transportation problems must be addressed. In the future, growing these educational forums should be a top priority. In addition, partnerships with local health authorities and non-governmental organisations can enhance resource availability and provide expert-led sessions, ensuring that the information shared is both accurate and relevant. Digital tools can be used to reach a wider audience and provide remote access, especially in resource-limited places. Furthermore, extending assistance and monitoring could prolong the positive effects of these initiatives in the long run. Globally combating malnutrition and promoting healthier, better-informed communities might be greatly aided by scaling up this concept throughout Rwanda and adapting it for use in other places in the world.
